# Bovine neonatal microbiome origins: a review of proposed microbial community presence from conception to colostrum

**DOI:** 10.1093/tas/txad057

**Published:** 2023-05-27

**Authors:** Riley D Messman, Caleb O Lemley

**Affiliations:** Department of Animal and Dairy Sciences, Mississippi State University, Mississippi State, MS 39762, USA; Department of Animal and Dairy Sciences, Mississippi State University, Mississippi State, MS 39762, USA

**Keywords:** bovine, developmental programming, inoculation, microbiome, neonate

## Abstract

In recent years, there has been an influx of research evaluating the roles of the reproductive tract microbiota in modulating reproductive performance. These efforts have resulted in a breadth of research exploring the bovine reproductive tract microbiota. The female reproductive tract microbiota has been characterized during the estrus cycle, at timed artificial insemination, during gestation, and postpartum. Additionally, there are recently published studies investigating in-utero inoculation of the bovine fetus. However, critical review of the literature to understand how the microbial shifts during a dam’s lifecycle could impact neonatal outcomes is limited. This review demonstrates a consistency at the phyla level throughout both the maternal, paternal, and neonatal microbiomes. Moreover, this review challenges the current gestational inoculation hypothesis and suggests instead a maturation of the resident uterine microbiota throughout gestation to parturition. Recent literature is indicative of microbial composition influencing metabolomic parameters that have developmental programming effects in feed utilization and metabolic performance later in life. Thus, this review enumerates the potential origins of neonatal microbial inoculation from conception, through gestation, parturition, and colostrum consumption while introducing clear paucities where future research is needed to better understand the ramifications of the reproductive microbiome on neonates.

## Introduction

Within the last decade, there has been a massive influx of bovine reproductive microbiome research introduced to the literature. The complex nature of biome data combined with differing interpretations has led to numerous questions regarding the role and relevance of the bovine reproductive tract microbiome. Authors agree the potential for microbial communities to modulate fertility within the dam exists, with recently published articles focused on eliciting mechanisms by which these fertility modulations occur (Srinivasan et al., 2021; Adanane & Chapwanya, 2022). However, literature exploring how the pre-existing proposed inoculation times affect the developing conceptus is minimal. Thus, this review will focus on literature proposing microbial communities and their roles during the stages of conceptus development. Additionally, the physiological insult of parturition on both neonatal and maternal microbiomes will be explored to address paucities within the literature, and to discuss how passive transfer through colostrum and environmental factors can contribute to the establishment of microbial communities. The objective of this review is to holistically approach the current literature to identify limitations and key connections that could provide direction for future research within the field of bovine reproduction.

Authors would like to acknowledge that this area of research is fairly novel in livestock. Thus, the studies incorporated in this review are pioneering livestock microbiome collection methods, analysis, and interpretations. In human literature, a uterine (Garcia-Grau et al., 2019), placental (de Goffau et al., 2019), or amniotic (Lim et al., 2018) microbiota in healthy women has still not been confirmed. This review acknowledges the limitations of the livestock current literature but reiterates the importance to highlight current results to drive future research.

## Conception

### The Maternal Microbiome

The female reproductive tract is the site of copulation, sperm deposition, fertilization, embryogenesis, and gestation within the bovine. Thus, commensal microbiota must be considered when evaluating inoculation of the growing conceptus. Researchers have been working to associate microbial composition within the female reproductive tract to fertility (Ault et al., 2019; Deng et al., 2019; Messman et al., 2020). These characterization studies have produced data representative of a dynamic healthy reproductive tract microbiota, and shown clear markers for dysbiosis, such as decreased diversity and loss of heterogeneity (Galvao et al., 2019). Recent characterization research has primarily focused on the vaginal and uterine microbiota; thus, these biomes will be the focus for potential female microbial contributions during conception.

Swartz et al. (2014) drove further research in the field by comparing human vaginal microbiota to bovine and revealing a stark contrast. Humans have a *Lactobacillus* spp. dominated reproductive tract microbiome; the lactic acid produced creates an acidic environment (pH < 4.5) that is effective in preventing pathogen colonization (Stout et al., 2020). However, the bovine vaginal environment has a near neutral pH (7.3) creating an environment where the phyla Bacteroidetes, Fusobacteria, and Proteobacteria dominate (Swartz et al., 2014).

Due to the vulva lying directly ventral to the anus, contamination within the vaginal tract with fecal material is highly likely. The cattle fecal microbiota is dominated by Firmicutes, Bacteroidetes, and Proteobacteria, respectively (Minseok and Wells, 2015). This clear overlap in shared phyla demonstrates the role of fecal contamination in the establishment of a residential vaginal microbiome, but the consistent presence of *Fusobacteria* and *Tenericutes* at varying ratios is noteworthy.

*Fusobacteria* spp. are gram negative, nonspore forming, obligately anaerobic (survive in low O2 environments > 8%) bacilli that are ubiquitous in the oral cavities of humans and animals (Brennan and Garrett, 2019). *Fusobacteria* spp. are mutualists within oral biofilms playing a role in structural support and binding secondary colonizers (Kolenbrander et al., 2010). Thus, the *Fusobacteria spp.* present within the vaginal tract could play similar role in biofilm formation, but an interesting linkage between *Fusobacteria nucleatum* and preterm delivery, still birth, and late term abortions has been recently proposed within human literature.

There is an association between periodontal disease in pregnant women and preterm delivery (Offenbacher et al., 1996). The proposed theory suggests transient bacteriemia, due to chronic periodontal disease, combined with an increase in blood flow to the uterus during pregnancy drives hematogenous spread of bacteria to the placenta resulting in fetal inoculation during late gestation (Meschia, 1983; Han et al., 2004). *F. nucleatum* is a causative agent of periodontal disease in humans and the bacteria has been identified in amniotic fluid of 10% to 30% of women preterm labor (Hill et al., 1998). To strengthen this association, Han et al. (2004) intravenously injected pregnant mice during late gestation with *F. nucleatum* to evaluate fetal outcomes. The injection resulted in preterm birth (entire litter stillborn) or full-term delivery with live (nonviable) and dead fetuses. *F. nucleatum* was isolated from injected mice’s placentas, amniotic fluids, and fetuses after delivery (Han et al., 2004). This study is notable because it introduces potential pathogenic roles of *Fusobacteria spp.* can cause during gestation. A Fusobacteria and Bacteroidetes dominated reproductive tract microbiota were associated with cows that eventually developed reproductive disease; these phyla synergistically cause reproductive disease via virulence and growth factor expression (Ong et al., 2021). Deeper metagenomic sequencing evaluating the species presence and virulence factors expressed is needed to determine Fusobacteria’s exact role within the bovine vaginal microbiota, but potential for uterine contamination as an opportunistic pathogen and subsequent negative consequences to fertility should be considered.

Tenericutes are considered commensal bacteria within the lower reproductive and urogenital tract, but their presence within the uterine environment can lead to adverse reproductive outcomes (Santos et al., 2011; Ong et al., 2021; Adanane and Chapwanya, 2022). The genera of *Ureaplasma* and *Mycoplasma* spp. are of particular interest as they lack a cell wall and typically exist within mammalian biomes as opportunistic pathogens (Santos Jr. et al., 2021). Moreover, these genera can be sexually transmitted between animals via secretions, semen, seminal plasma, and preputial and vaginal mucus (Miller et al., 1994). Thus, bacteria transfer is of concern when using assisted reproductive technologies (Crane and Hughes, 2018).

*Ureaplasma diversum* has been shown to cause granular vulvovaginitis syndrome in female cattle. This infection causes acute inflammation within the reproductive tract, tissue damage, and decreased fertility (endometritis, spontaneous abortion, early embryonic death; Nascimento-Rocha et al., 2017; Santos Jr. et al., 2021). *U. diversum* is commonly isolated within placental tissue, lungs, and abomasal fluid of late abortion fetuses and neonates postmortem (Gagea et al., 2006; Anderson, 2007). Thus, the presence of Tenericutes at high ratios within the vaginal microbiome of cows should be considered a risk factor for subsequent infection within the dam or fetus. However, no physiological mechanism by which *Ureaplasma* or *Mycoplasma* spp. proliferate within a eubiotic system has been elicited.

**Figure 1.**
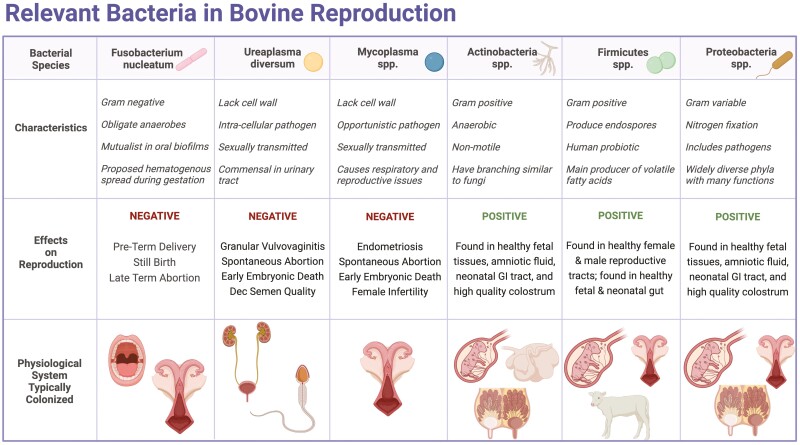
Table depicting bacterial species that are relevant within bovine reproductive physiology. This table includes important characteristics, documented effects of the bacteria on reproduction, and the physiological system that is typically colonized within the body. Literature cited can be found within the text. Created with BioRender.com.

Previous research hypothesized Tenericutes ascend from the vaginal tract to uterine body during estrus, or artificial insemination (AI), when the cervix is dilated (Santos Jr. et al., 2021). *U. diversum* is an obligate intracellular pathogen capable of infecting endometrial cells and altering prostaglandin production by increasing PGF2a production and decreasing PGE2 (Kim et al., 1994). These studies provide evidence that Tenericutes have the potential to affect fertility via both virulence and alterations of hormone concentrations with the uterine environment. To conclude, the presence of these opportunistic pathogens within vaginal biofilms should be further explored, especially regarding infertility or persistent infections within female cattle. Figure 1 shows relevant bacteria within bovine reproduction that could be attributed to both negative and positive outcomes.

### The Paternal Microbiome

The paternal microbiome is often overlooked in female reproductive microbiome research. However, the spread of bacteria from the male to female through coitus or within the ejaculate is well-reported (Givens, 2008). Thus, implications of introduction of the native male biome to the female reproductive tract should be further explored regarding colonization, immune response, and fertility.

Wickware et al. (2020) characterized the epithelial surface microbiome of the penis and prepuce in 92 healthy postpubertal bulls via 16S rRNA sequencing. The dominant phyla within the bull penis/prepuce included Firmicutes, Fusobacteria, Bacteroidetes, Proteobacteria, and Actinobacteria. These phyla overlap with dominant phyla within both the cow vaginal tract and fecal matter (Swartz et al., 2014; Minseok and Wells, 2015; Wickware et al., 2020). Authors concluded the soil, feces, urine, and cow vagina likely contribute to the epithelial surface biome of the penis and prepuce in the bull (Wickware et al., 2020). These conclusions further implicate the role of the environment and nutrition (feces produced) in colonization of the bovine reproductive tract.

Moreover, the microbial composition of bull ejaculates is of interest considering the site of semen deposition is the uterine body in cattle AI. Due to the introduction of AI, semen collection and processing techniques introduce a secondary source of ejaculate contamination (Sannat et al., 2015). To compensate for bacterial contamination, antibiotics are commonly added to cryopreserved ejaculate, but bacteria can still be isolated from thawed bull semen samples (Zampieri et al., 2013). The presence of bacteria within an ejaculate does not equate infection or decrease sperm quality (Baud et al., 2019), but the effects of bacteria within the uterine environment of the female is unknown. Cojkic et al. (2021) evaluated ejaculates from healthy Holstein bulls (*N* =18); ejaculates were collected, extended, and frozen in liquid nitrogen until DNA extraction for 16S rRNA analysis. The most common genera found in the ejaculates included *Porphyromonas*, *Fusobacterium*, and *Ruminococcaceae*; these findings agree with Wickware et al. (2020) showing overlap in phyla presence within the penis/prepuce and the ejaculates of bulls (Cojkic et al., 2021). Despite an increase in research focused on associating male fertility with the paternal microbiome, there is limited literature evaluating effects of the paternal microbiome colonization in the maternal reproductive can impact the conceptus during gestation.

### The Embryonic Microbiome

In summation, the maternal and paternal reproductive microbiomes likely contribute to the uterine environment pre and postconception. The roles of specific microbes in modulating fertility are not well-established, but bacteria, such as *U. diversum*, is capable of attaching to sperm and endometrial cells (Lingwood et al., 1990). Thus, the likelihood of microbes within the ampullary-isthmus junction during conception is likely. To the authors’ knowledge, microbial characterization research within the bovine oviduct has not been performed to date.

Exposure to microbes during the embryonic stage is not well-defined in bovine research. In cryopreserved embryos, both bacterial and fungal isolates were found, but the relevance of these findings to in-vivo conception is negligible (Bielanksi et al., 2003). Moreover, contamination of in-vitro fertilization culture media is associated with negative outcomes for the embryo (Borges & Vireque, 2020). However, the central dogma of a sterile environment within the reproductive tract is controversial due to the development of advanced culture independent methodologies such as 16S rRNA sequencing, shotgun sequencing, and metagenomic sequencing (Perez-Muñoz et al., 2017; Wang et al., 2022). An interesting hypothesis within recent human literature is that microbial populations within the oviduct could have epigenetic effects on the embryo (El Hajj and Haff, 2013). Specifically, bacterial pathogens can be considered epimutagens that can reshape genomes to cause lasting effects within the embryo (Bierne et al., 2012; Borges and Vireque, 2020). Thus, the susceptibility of embryos and the maternal environment to microbial modulations poses an interesting hypothesis, that the microbial environment is integral in conceptus programming starting at fertilization.

## Gestation

Historically, the mammalian conceptus was regarded as sterile with the placenta serving as a physiological barrier preventing microbial colonization (Escherich, 1886; Funkhouser and Bordenson, 2013). However, decades of reproductive physiology research have revealed the likelihood of a completely sterile uterine environment during the mammalian pregnancy is low. Pregnancy modulates the maternal circulatory system, specifically there is an increase (30% to 40%) in blood volume resulting in increased cardiac output (30%) and increased blood flow (16%) to the uterus during late gestation due to increased nutrient demand of the growing conceptus (Rosenfeld, 1984; Vonnahme et al., 2013). Increased blood flow also increases delivery of circulating hormones, cytokines, metabolites, and microbes to the placental vascular bed (Rosenfeld, 1984; Hsu & Nana, 2014). Current literature supports the theory of microbial inoculation of the uterus via hematogenous route in postpartum dairy cattle (Jeon et al., 2017), pregnant mice (Fardini et al., 2010), and humans (Katz et al., 2009). Thus, sterility in the uterus throughout gestation is improbable, but the question remains of the inoculating microbe’s role in conceptus development.

**Figure 2.**
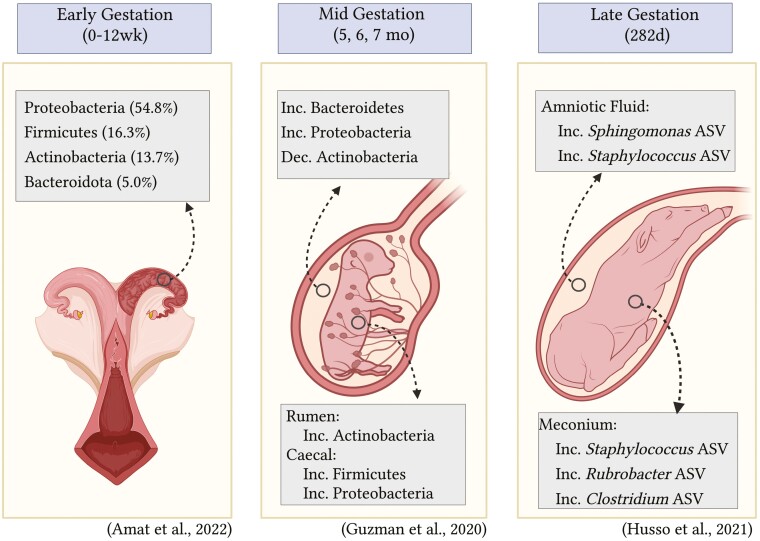
Diagram of the bovine reproductive tract during early, mid, and late gestation with common bacterial species and where they are located within both the uterus and the fetus. Created with BioRender.com.

Within human research, the initial microbial colonization of the neonate is considered the most important determinant of future host-microbe interactions that can modulate an individual’s risk for noncommunicable disease (Collado et al., 2016). Thus, current bovine microbiome research has begun to explore the microbial presence during gestation to investigate the critical role of micro-organisms in fetal development (Amat et al., 2022a; Hummel et al., 2022). Throughout gestation, the major physiological changes that are occurring in the dam, growing conceptus, and uterine environment attributes to the potential for conceptus exposure to a dynamic community of micro-organisms throughout gestation (Figure 2).

### Early Gestation

A recent study by Amat et al. (2022b) characterized the relative abundance and microbiome composition in the amniotic fluid (1855 OTU), allantoic fluid (2704 OTU), intestine (1323 OTU), and placental cotyledonary tissue (1347 OTU) in 83-d-old calf fetuses collected via ovariohysterectomy. Interestingly, only 55 OTU were shared within these tissue samples, but the overlap represented the dominating phyla of bacteria (Amat et al. 2022b). These characterizations indicate an overall dominant microbial community within the reproductive tract, but OTU fluctuations within individual biomes is highly likely. A diverse and relatively unique microbial population was found in all four fetal samples. Within all samples, Proteobacteria (54.8%), Firmicutes (16.3%), and Actinobacteriota (13.7%) were the most abundant (Amat et al. 2022b). Interestingly, these microbial populations are consistent with the common phyla found within the vaginal microbiota (Swartz et al., 2014; Ault et al., 2019; Messman et al., 2020). The presence of *Actinobacteriota* was greater in the intestine (5.5%) and placenta (3.2%) compared to the allantoic (0.5%) and amniotic (1.0%) fluids (Amat et al. 2022b). Authors hypothesized that there is a unique microbiota between fetal intestine, placenta, and placental fluids due to differences in physiological, biochemical, and immunological properties that vary between sites (Amat et al. 2022b). This observation is consistent with general microbial principles that discuss microbial preferences for a certain environment based on species, strain, and function (Keller and Zengler, 2004). The observation of distinct microbial communities this early in gestation, indicates an even earlier inoculation timepoint. Interestingly, similar microbial populations were identified in calves harvested later in gestation. This leads authors to question if the microbiome is developing with the conceptus instead of inoculating at a specific timepoint.

### Mid Gestation

In a study by Guzman et al. (2020), calf tissues were harvested at 5 (*N* = 4), 6 (*N* = 4), and 7 (*N* = 4) mo of age after slaughter of dam. Briefly, the uterus, with the placenta and fetus, was removed 35 to 45 min postslaughter; all tissue samples (amniotic fluid, meconium, ruminal fluid, ruminal tissue, cecal fluid, and cecal tissue) were collected within the abattoir utilizing consistent and sterile techniques. Contamination control samples were cross referenced to account for contamination through the collection and analysis process. In total, 559 bacterial exact sequence variants (ESVs) and 1736 archaeal ESVs were identified. Across all samples, Proteobacteria (32%), Firmicutes (31%), and Actinobacteria (26%) were the most dominant bacterial phyla; Euryarcheota (88%), Crenarchaeota (6%), and Kararcheota (5%) were the dominant phyla within the archaeal ESVs (Guzman et al., 2020). Moreover, the dominant phyla in the amniotic fluid were different than the calf gastrointestinal tract tissues; this is consistent with the findings in 83-d-old calves (Amat et al. 2022b). Guzman et al. (2020) also demonstrated a temporal change from 5 to 7 mo of gestation within the abundance of fetal calf microbial communities. Together these suggest the fetal calf’s gastrointestinal microbiome diverges from that of the amnion during gestation, and there are well-established and distinct microbial communities within different fetal sites by 5 mo of gestation.

This study contributes to the building literature that refutes the sterile womb hypothesis (Guzman et al., 2020; Husso et al., 2021; Amat et al. 2022b; Bolte et al., 2022). Interestingly, research is primarily focused on determining the inoculation timepoint, but as this review has demonstrated micro-organisms are present within the reproductive tract prior to conception. Thus, future research should focus on how the physiological shifts in environmental conditions within the reproductive tract during gestation can impact microbial growth. Unfortunately, the current literature most commonly highlights phyla. Taxonomic identification to the phyla level is considered more reliable but fails to highlight specific characteristics of bacteria living within that environment. Thus, there is likely physiological restraints (pH, oxygen availability, nutrient sources, etc.) that prevent similar species from colonizing within these locations leading to the microbial discrepancies between fetal and placental sites.

### Late Gestation

A recent study by Husso et al. (2021) evaluated the amniotic fluid and meconium microbiome in full-term Belgian Blue calves (*N* = 23) that were delivered via Cesarean section. Interestingly, the amniotic fluid samples had similar (*P* = 0.17) 16S rRNA gene copies as controls albeit the OTU identified were different; authors commented that it is likely this low microbial biomass has little impact. Moreover, the amniotic and meconium microbial profile was dominated by Proteobacteria, Firmicutes, Bacteriodetes, and Actinobacteria in agreement with the previous studies in early gestation (Guzman et al., 2020; Amat et al. 2022b). Moreover, only 5 of 24 meconium samples were successfully cultured, and all amniotic fluid samples were culture negative. While culture limitations are clearly defined, one could inquire about the physiological status (dead vs. alive) of the bacteria identified using the 16S technique.

### Gestational Inoculation vs. Maturation Hypothesis

Upon review of key studies identifying bovine fetal inoculation throughout gestation, there are interesting similarities that warrant further discussion. Firstly, all these studies throughout gestation were conducted in different countries (United States, Australia, and Belgium) using different collections techniques (ovariohysterectomy, slaughter/harvest, and Cesarean section) and varying levels of contamination controls (Guzman et al., 2020; Husso et al., 2021; Amat et al. 2022b). However, despite these differences the microbial populations identified in the amnion and fetal gut (rumen/small intestine tissue) were similar at the phyla level. This suggests a commensal microbial population present in the reproductive tract during gestation. Interestingly, there was a decrease in microbial abundance (OTU/ESV/ASV) throughout bovine gestation with little notable microbial abundance close to parturition (Husso et al., 2021). This leads authors to speculate microbial inoculation throughout gestation may be less likely compared to microbial maturation.

Overlap was identified in dominant phyla (Proteobacteria, Bacteroidetes, Firmicutes, and Actinobacteria) found within the uterine and vaginal tract of open cows (Swartz et al., 2014; Messman et al., 2020) as well as in the epithelial surface microbiota of the bull penis/prepuce and semen (Wickware et al., 2020). Thus, it is highly likely these bacteria are present in the uterine body prior to conception, embryo migration, and implantation. After conception, the cervix is a formidable barrier with mucosa-lined cartilaginous rings that become tightly convoluted under progesterone influence (Bondurant, 1999). Thus, authors hypothesize micro-organisms detected throughout gestation are present prior to conception and those identified are capable of surviving the physiological changes in the uterine environment during gestation.

Likely, the bacteria that fall in the phyla of Bacteriodetes, Proteobacteria, and Firmicutes can survive in harsh conditions with low nutrients and oxygen availability; thus, their presence in the fetal gut (rumen/small intestine tissue) and meconium is due to survival capabilities. Moreover, the bacteria within the Actinobacteria phyla are identified within the gut early in gestation, but there is an increased prevalence of this phyla within the amniotic fluids as gestation persists (Guzman et al., 2020; Husso et al., 2021; Amat et al. 2022b). This is interesting and poses a question about Actinobacteria’s ability to survive within these environments. If this phylum lacks attachment capabilities within the fetus, these bacteria (dead or alive) would be shed into the amniotic fluid and then routinely swallowed during the last month of gestation (Gilbert & Brace, 1989). During the movement of Actinobacteria through the gastrointestinal tract of the fetus, other microbes in dominating phyla could be utilizing these dead microbes as a source of protein and energy. Thus, this would explain the decrease in specific phyla abundance throughout gestation. To support this hypothesis further, the only sequencing technique utilized is 16S rRNA gene amplification which does not discriminate between dead or alive bacteria (Li et al., 2017). Thus, while these phyla have been characterized, their metabolic status (dormant, live, dead, nongrowing) cannot be determined. Together, these observations indicate microbial findings throughout gestation could be attributed to the maturation and survival of the commensal uterine/vaginal/paternal microbial communities are present at conception due to the closing of the cervix and their inability to leave the system. While inoculation is still probable and has been demonstrated in humans (Bolte et al., 2022), it is less likely all detected micro-organisms originated from an inoculation route and more plausible they were within that environment before pregnancy establishment. Future research exploring this hypothesis is needed and could help elucidate some discrepancies within the growing field of research.

### Programming Effects of the Gestational Microbiome

Few studies have examined prenatal programming of the microbiome in ruminant species. For example, maternal environmental manipulations are a promising area of research in relation to improving offspring production outputs; however, more controlled studies are needed in this area. Another important area of research is linking the microbiome to host metabolome profiles, which can alter important phenotypic traits. Elolimy et al. (2019) examined maternal rumen-protected methionine supplementation during the last 28 d of gestation on offspring fecal microbiota and metabolome as well as growth performance. For this study, maternal methionine supplementation increased heifer calf size at birth, while measures of beta and alpha diversity of fecal microbial communities were similar at birth. However, shifts in specific bacterial taxa of the hindgut and fecal metabolome were observed. This carried over into the preweaning phase whereby offspring born to methionine supplemented dams had increased *Ruminococcus* and *Fusobacerium*, which have been linked to volatile fatty acid production in the hindgut (Elolimy et al., 2019). Of great interest, this study showed that maternal supplementation during late gestation can shift offspring microbiota and metabolome to a more efficient profile, such as decreasing pathogens and enhancing production of vitamins.

In a recent study with woman carrying twin pregnancies, researchers examined 150 pairs of twin neonates to explore gut microbial communities and their metabolic profiles in relation to indicators of fetal growth restriction (Yang et al., 2022). Interestingly, early neonatal gut microbiota diversity was positively correlated with the severity of fetal growth restriction and an adverse intra-uterine environment was associated with neonatal gut microbiota dysbiosis, which was more pronounced in monochorionic-diamniotic twins vs. dichorionic-diamniotic twins. Specifically, in monochorionic-diamniotic twins with fetal growth restriction researchers observed increased *Coprococcus*, *Robinsoniella*, and *Oscillospira* and decreased *Acinetobacter*, *Enterococcus*, and *Actinobacillus*. Similar to changes in gut microbiota, metabolic meconium and fecal profiles were more dissimilar in the monochorionic-diamniotic twins with fetal growth restriction, whereby smaller twins had decreased concentrations of cysteine, methionine, and dipicolinic acid (Yang et al., 2022). These researchers suggested decreased abundance of neonatal *Enterococcus* and *Acinetobacter* may be linked to lowered fecal concentrations of methionine and cysteine. These metabolic changes in methionine are especially interesting as maternal methionine supplementation during late pregnancy shifted microbiota and metabolome profiles during the neonatal period of calves (Elolimy et al., 2019). Specifically, the microbiota from methionine treated animals were enriched with a greater number of functional genes involved in methionine and cysteine metabolism and a lower number of functional genes for glycine, serine, and threonine metabolism (Elolimy et al., 2019). Interestingly, apart from early immune function and neonatal growth, the maternal gut microbiome in mice has been linked to fetal neurodevelopment (Vuong et al., 2020), which is an area that needs to be further explored in livestock species.

Innovative studies have characterized changes in neonatal gut microbial communities in models of fetal growth restriction or maternal late pregnancy supplementation strategies, however, fewer studies have linked causative roles of the maternal gut microbiome with compromised pregnancies. Researchers have examined contributions of the maternal gut microbiome and targeted metabolomics to pre-eclampsia, a leading cause of placental dysfunction resulting in greater risk of maternal and perinatal morbidity. Importantly, maternal gut microbial dysbiosis may contribute to the development of pre-eclampsia, while *Akkermansia muciniphila* was shown to regulate vascular placental remodeling through propionate and butyrate metabolites in pre-eclamptic rats (Jin et al., 2022). Therefore, maternal microbial and metabolome panels may reveal potential biomarkers for pre-eclampsia risk or other forms of compromised pregnancies which are more relevant to livestock.

### Perinatal Calf Microbiome

There is a breadth of literature evaluating the postpartum neonatal microbiota in comparison to published literature evaluating the dam reproductive tract microbiota during gestation. Shockingly, despite differences in breed, location, time after birth, and sampling site, there is a consistent gastrointestinal microbiota in calves dominated by Proteobacteria, Firmicutes, Actinobacteria, and Bacteroidetes (Gomez et al., 2017; Alipour et al., 2018; Barden et al., 2020; Fan et al., 2021; Zhu et al., 2021; Woodruff et al., 2022). These, again, overlap with the early (Amat et al. 2022b), mid (Hummel et al., 2022), and late (Guzman et al., 2020) gestation microbiota identified. Moreover, these are the similar biomes to the dam reproductive tract (Swartz et al., 2014) and male reproductive tract (Wickware et al., 2020). This persistent overlap should be noted, but although the dominant phyla remain the same there is clear shifts within the neonate after parturition.

In a study by Alipour et al. (2018), the feces from calves were evaluated at 0 h, 24 h, and 7 d; these samples were also compared to the dam feces, mouth, and vaginal microbiota. Notably, there was a drastic change in fecal microbiota from 0 to 24 h of age, where there was an increase in *Escherichia Shigella, Clostridium sp,* and *Enterococcus sp.* (Alipour et al., 2018). From 24 h to 7 d, the calf rectal microbiota began to resemble the dam demonstrating an establishment of residential microbiome was primarily composed of *Faecalibacterium, Bacteroides, Lactobacillus,* and *Butyricicoccus* (Alipour et al., 2018). Authors associated the drastic differences between the 0 h (meconium) from the 24 h sample is due to the in-utero colonization of the gut, this microbial population is shed in the meconium and environmental colonization of the neonatal gut could occur (Alipour et al., 2018). On day 7, the abundance of bacteria in the rectum increased but there was a decrease in species richness. This decrease in diversity is consistent with microbial dysbiosis within the gastrointestinal tract (Wilkins et al., 2019). In studies evaluating diarrhea instances in calves, the fecal microbiome has a decreased richness and abundance compared to healthy animals; moreover, Actinobacteria was decreased in diarrheic calves (Gomez et al., 2017) and *Lactobacillus sp.* were associated with healthy calves (Fan et al., 2021). In the study by Alipour et al. (2018), the dominating genera at 24 h are consistent with calves that develop diarrhea between 21 and 35 d (Fan et al., 2021); thus, it could be hypothesized calves that without the proper gut microbiome development by 7 d of age could be more susceptible to neonatal diarrhea and persistent gastrointestinal dysbiosis.

### Colostrum Microbiome

When considering gut recolonization, nutrition should be the first factor evaluated. Thus, the colostrum microbiota could be indicative of neonatal dysbiosis outcomes. Again, colostrum is dominated by the same four phyla Firmicutes, Bacteriodetes, Proteobacteria, and Actinobacteria (Lima et al., 2017; Chen et al., 2021; Van Hese et al., 2022). In one study, the genera *Actinobacter* comprised 16.2% and *Lactococcus* comprised 4.0% of the colostrum microbiota (Van Hese et al., 2022), these genera fall, respectively, within the Actinobacteria (Gomez et al., 2017) and Lactobacillus (Fan et al., 2021) phyla that have been associated with gastrointestinal health in calves.

Throughout this review, the phyla Actinobacteria has been reported modestly in many articles. Specifically, it is typically the fourth most abundant phyla in healthy animals. During late gestation, Actinobacteria is commonly isolated from the amniotic fluid (Guzman et al., 2020; Husso et al., 2021), and Gomez et al. (2017) demonstrated a positive relationship with Actinobacteria and health in neonatal calves. Notably, genus within this phylum, including *Actinobacter*, has been identified in high-quality colostrum samples (Van Hese et al., 2022). Actinobacteria are gram positive, nonmotile, anaerobic, branching rods (Belizario et al., 2015). In humans, the family Bifidobacteria is the most represented in the gut (Binda et al., 2018) and neonates born via Cesearean section had significantly less abundance of Actinobacteria during their first week of life (Dogra et al., 2015), 1 mo (Huurre et al., 2008), and 3 mo(Kabeerdoss et al., 2013). Interestingly, neonates that were breastfed had a higher abundance of Actinobacteria within the gut than formula fed neonates (Bezirtzoglou et al., 1997). Postweaning in humans also causes a decrease in the Actinobacteria families (Bifidobacteria) within the gastrointestinal tract (Ley et al., 2006).

The inconsistent presence of Actinobacteria throughout conceptus development is intriguing. In humans, this phylum has been shown to maintain intestinal barrier functions (Ashida et al., 2011), biodegradation of resistant starches (Ryan et al., 2006), decrease inflammatory responses (O’Mahony et al., 2005), and has antidepressant properties due to increase tryptophan production (Desbonnet et al., 2009; Binda et al., 2018). Tryptophan, the sole precursor of serotonin, has been highlighted for its role in neurotransmission, neuroendocrine actions, and intestinal immune responses which subsequentially alter the brain-gut axis (Gao et al., 2020). Together, these provide major implications for this phylum in modulating the bovine neonatal gastrointestinal microbiota and subsequent health outcomes. Evaluation of this phylum in colostrum samples in correlation with neonatal morbidity incidence could be beneficial in elucidating its importance in calf gut colonization.

### Summary

To summarize, throughout this review it has become evident that the bovine reproductive tract, developing gut, and colostrum are dominated by Proteobacteria, Firmicutes, Bacteroidetes, and Actinobacteria. Moreover, microbial presence, from both maternal and paternal origins, within the reproductive tract prior to conception is likely. The presence of microbes can contribute to developmental programming of the conceptus through inoculation and modulations of the uterine environment. While in-utero microbial inoculation of the conceptus via hematogenous route is certainly possible, it is more likely that microbes are already present and capable of survival. Contributions to the neonatal gut microbiome is multifactorial, with inoculation from in-utero exposure, vaginal tract during parturition, dam interactions, external environment, and colostrum are all likely. However, the role of Actinobacteria within this colonization should be further explored due to the numerous beneficial roles that have been demonstrated within human physiology. Lastly, the efforts to identify the microbial origins within a developing conceptus have clearly shown a stable residential microbiome, and future research exploring induced dysbiosis could yield data that lead to a better understanding of these bacteria’s roles within the bovids.
